# Tissue Transglutaminase Promotes Drug Resistance and Invasion by Inducing Mesenchymal Transition in Mammary Epithelial Cells

**DOI:** 10.1371/journal.pone.0013390

**Published:** 2010-10-12

**Authors:** Anupam Kumar, Jia Xu, Samuel Brady, Hui Gao, Dihua Yu, James Reuben, Kapil Mehta

**Affiliations:** 1 Department of Experimental Therapeutics, The University of Texas M. D. Anderson Cancer Center, Houston, Texas, United States of America; 2 Department of Molecular and Cellular Oncology, The University of Texas M. D. Anderson Cancer Center, Houston, Texas, United States of America; 3 Department of Hematopathology, The University of Texas M. D. Anderson Cancer Center, Houston, Texas, United States of America; 4 Graduate School of Biomedical Sciences, The University of Texas Health Science Center, Houston, Texas, United States of America; East Carolina University, United States of America

## Abstract

Recent observations that aberrant expression of tissue transglutaminase (TG2) promotes growth, survival, and metastasis of multiple tumor types is of great significance and could yield novel therapeutic targets for improved patient outcomes. To accomplish this, a clear understanding of how TG2 contributes to these phenotypes is essential. Using mammary epithelial cell lines (MCF10A, MCF12A, MCF7 and MCF7/RT) as a model system, we determined the impact of TG2 expression on cell growth, cell survival, invasion, and differentiation. Our results show that TG2 expression promotes drug resistance and invasive functions by inducing epithelial-mesenchymal transition (EMT). Thus, TG2 expression supported anchorage-independent growth of mammary epithelial cells in soft-agar, disrupted the apical-basal polarity, and resulted in disorganized acini structures when grown in 3D-culture. At molecular level, TG2 expression resulted in loss of E-cadherin and increased the expression of various transcriptional repressors (*Snail1, Zeb1, Zeb2* and *Twist1*). Tumor growth factor-beta (TGF-β) failed to induce EMT in cells lacking TG2 expression, suggesting that TG2 is a downstream effector of TGF-β-induced EMT. Moreover, TG2 expression induced stem cell-like phenotype in mammary epithelial cells as revealed by enrichment of CD44^+^/CD24^-/low^ cell populations. Overall, our studies show that aberrant expression of TG2 is sufficient for inducing EMT in epithelial cells and establish a strong link between TG2 expression and progression of metastatic breast disease.

## Introduction

Tumor cell resistance to systemic therapies and metastasis to distant tissues pose major impediment to successful treatment of breast cancer [1]. Therefore, understanding the molecular mechanisms that contribute to the development of drug resistance and facilitate metastasis could provide valuable targets for effective control and treatment of the disease. Transglutaminase 2 (TG2), a pro-inflammatory protein, has received considerable attention recently for its potential role in cancer cells. There is ample evidence supporting that metastatic and drug-resistant breast cancer cells express high basal levels of TG2 [2] and that its expression is associated with increased cell survival, invasion and motility of cancer cells [3] . However, the knowledge on how TG2 promotes these phenotypes remains elusive.

TG2 is a multifunctional protein implicated in diverse physiological and pathological processes [4]. In addition to transamidation activity, TG2 can catalyze GTPase [5], protein disulfide isomerase [6] and kinase activities [7]. In normal tissues, TG2 expression is upregulated in response to tissue injury and other stressors; there it plays a role in restoring the normal homeostasis [8]. In such pathological conditions as tissue fibrosis and cancer, TG2 expression within the cell or its microenvironment promotes cell adhesion and modulates intracellular signaling [9]. For example, intracellular TG2 is known to activate focal adhesion kinase (FAK), protein kinase B, and Akt [10], cyclic AMP response-element binding protein [11] , platelet-derived growth factor [12], and the nuclear factor-κB (NF-κB) [13] . In the extracellular environment, TG2 can modify the extracellular matrix proteins and alter cell-cell (homotypic) and cell-matrix (heterotypic) interactions [14]. Although, emerging lines of evidence suggest a close link between TG2, drug resistance, and metastasis of cancer cells, the pathways that contribute to these events remain largely unknown.

Recently epithelial-to-mesenchymal transition (EMT), which shares many molecular characteristics with cancer stem cells, has been implicated to play a role in cancer metastasis [15], [16]. EMT is a complex dynamic process that occurs during embryonic development for reprogramming of epithelial cells [16]. Its reactivation during adult life has been associated with pathological conditions, such as inflammation, fibrosis, and cancer [16], [17]. The EMT promotes the detachment of cancer cells from the primary tumor and facilitates migration via loss of cellular polarity and adhesion [17]. Moreover, emerging evidence suggests a strong link between EMT and chemoresistance and radioresistance [17], [18]. Therefore, understanding of molecular mechanisms that lead to EMT is important for designing novel therapeutic strategies for increasing drug sensitivity and the suppression of metastasis toward better treatment outcomes in cancer patients.

Here we demonstrate that aberrant expression of the pro-inflammatory protein, TG2, in mammary epithelial cells (MECs) is associated with loss of E-cadherin, cellular polarity, upregulation of mesenchymal markers, such as fibronectin, vimentin, N-cadherin and transcriptional repressors, such as *Snail1, Zeb1, Zeb2* and *Twist1.* Moreover, our data suggest that TG2 is a downstream mediator of tumor growth factor-beta (TGF-β)-induced EMT. We conclude that TG2 expression signals the onset of EMT in epithelial cells and contributes to their increased survival and metastatic potential and hence represents a promising therapeutic target for drug-resistant and metastatic breast cancer.

## Results

### TG2 induces EMT in mammary epithelial cells

**Figure 1:**
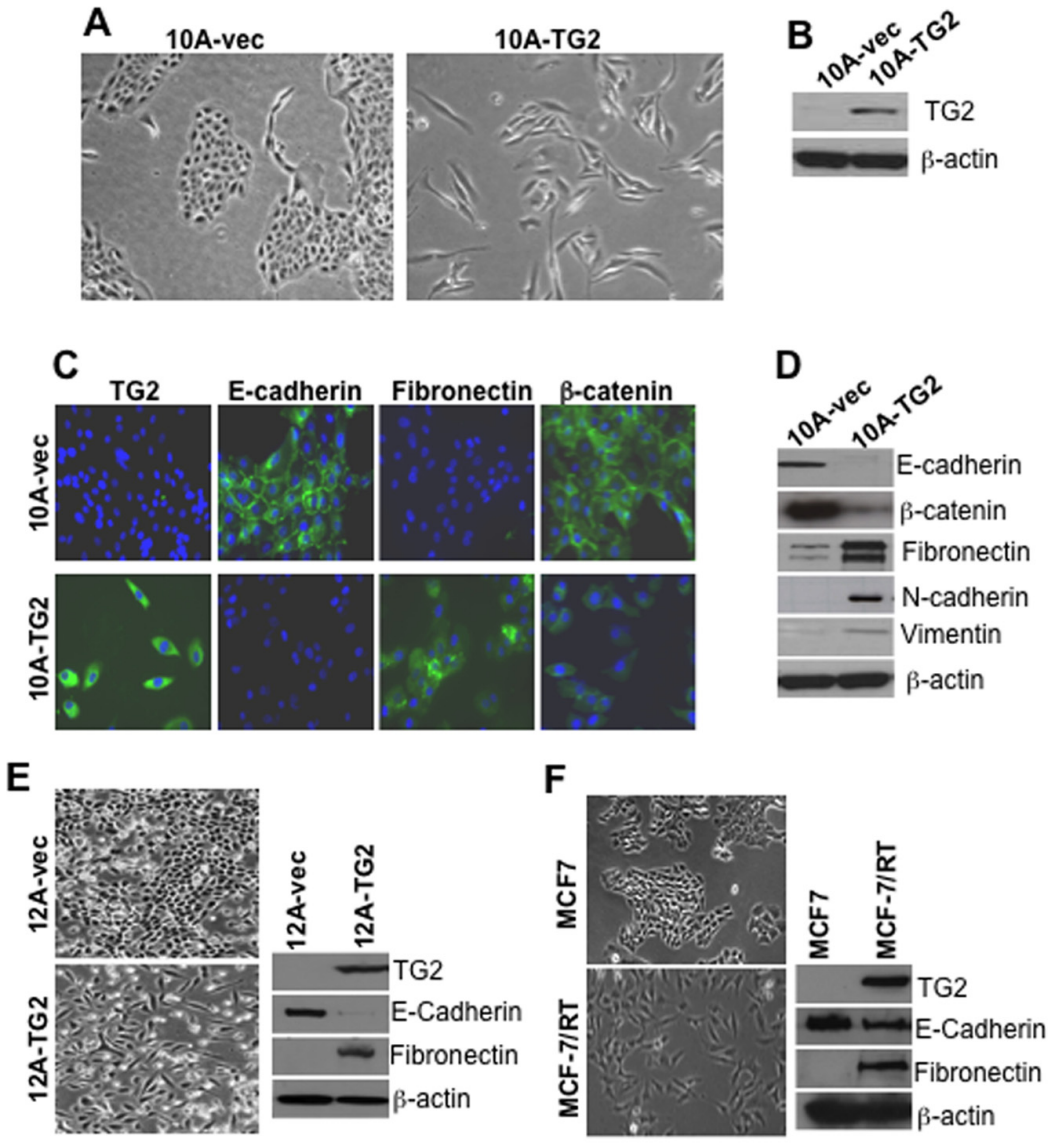
TG2 induces EMT in mammary epithelial cells. MCF10A and MCF12A cells were stably transfected with vector alone (10A-Vec and 12A-Vec) or lentiviral-TG2 construct (10A-TG2 and 12A-TG2). (**A**) Phase-contrast images of 10A-Vec and 10A-TG2 cells after 48 hr culture in medium. Magnification 10X. (**B**) Immunoblot showing relative expression of TG2 in 10A-Vec and 10A-TG2 cells. (**C**) Immunofluorescence due to TG2 and EMT markers in 10A-Vec (top panel) and 10A-TG2 (bottom panel) cells. Green fluorescence shows the expression and localization of indicated proteins and DAPI (blue) staining shows the nuclei. (**D**) Immunoblot analysis of the indicated EMT markers in 10A-Vec and 10A-TG2 cells. Expression of epithelial cell markers (E-cadherin, β-catenin) and mesenchymal cell marker (N-cadherin, fibronectin, and vimentin) was examined by immunoblotting. (**E**) Morphology (left penal) and immunoblot analysis of TG2, epithelial marker (E-cadherin) and mesenchymal marker (fibronectin) expression in 12A-vec and 12A-TG2 cells. (**F**) Morphology (left penal) and immunoblot analysis for constitutive TG2, epithelial marker (E-cadherin) and mesenchymal marker (fibronectin) expression in drug-sensitive and drug-resistant (RT) MCF-7 cells. Multiple stable polyclones were established from MCF10A and MCF12A cells and experiments were repeated multiple times with similar results using different clones.

We previously reported that metastatic breast cancer cells express high basal levels of TG2 [2] and that increased expression of TG2 in breast cancer cells contributes to their increased survival, invasion, motility and drug resistance [2], [3]. To understand the role of TG2 in metastatic transformation, we stably transfected TG2 into a non-transformed human breast mammary epithelial cell line MCF10A. TG2-overexpressing MCF-10A cells (10A-TG2) showed marked changes in their morphology compared to the vector-transfected (10A.Vec) cells. As shown in Fig. 1A, 10A-Vec cells appeared rounded with cobblestone epithelial morphology and grew as tightly connected clusters. 10A-TG2 cells, in contrast, displayed spindle-like shape and exhibited scattered distribution of fibroblast-like cells with disrupted cell-to-cell adhesion (Fig. 1A). Expression of TG2 in these cells was confirmed by immunoblotting (Fig. 1B) and immunostaining (Fig. 1C). The mesenchymal nature of 10A-TG2 cells was further confirmed by the accumulation of actin-stress fiber (Supplemental Fig. S1). These results suggested that TG2 expression in epithelial cells is associated with their transition into mesenchymal cells.

The morphological changes during EMT are driven by a number of molecular alterations, including loss or decrease of epithelial cell markers (e.g., E-cadherin and β-catenin) and *de novo* expression of mesenchymal markers (e.g., N-cadherin, vimentin, and fibronectin) [17]. Indeed, 10A-vec cells expressed high levels of E-cadherin and β-catenin (Fig. 1C and D) but low or undetectable levels of fibronectin (Fig. 1C and D), vimentin and N-cadherin (Fig. 1D). 10A-TG2 cells, in contrast, showed almost complete loss of E-cadherin and reduced expression of β-catenin (Fig. 1C and D). Similarly, the expression of fibronectin (Fig. 1C and D), N-cadherin and vimentin (Fig. 1D) was upregulated in 10A-TG2 cells. 10A-vec cells showed membranous staining of E-cadherin and β-catenin but no staining for fibronectin (Fig. 1C). In contrast, 10A-TG2 cells showed loss of E-cadherin staining, reduced cytoplasmic β-catenin and increased fibronectin staining (Fig. 1C). To rule out that TG2-induced EMT is not unique to MCF10A cells, we also determined the effect of TG2 expression in another non-transformed human breast mammary epithelial cell line, MCF12A. Results shown in Figure 1E revealed similar changes in the morphology, E-cadherin, and fibronectin expression in response to TG2 in these cells. Moreover, drug-resistant MCF-7/RT cells, which express high constitutive levels of TG2 [19], exhibited similar fibroblast-like morphology, down regulation of E-cadherin and gain of fibronectin expression when compared with their TG2-deficient, drug-sensitive counterpart MCF-7 cells (Fig. 1F). These results implied that TG2 expression results in the loss of cell-cell adhesion by inducing EMT in mammary epithelial cells.

### TG2 expression is associated with increase in Snail1, Twist1 and Zeb1

**Figure 2:**
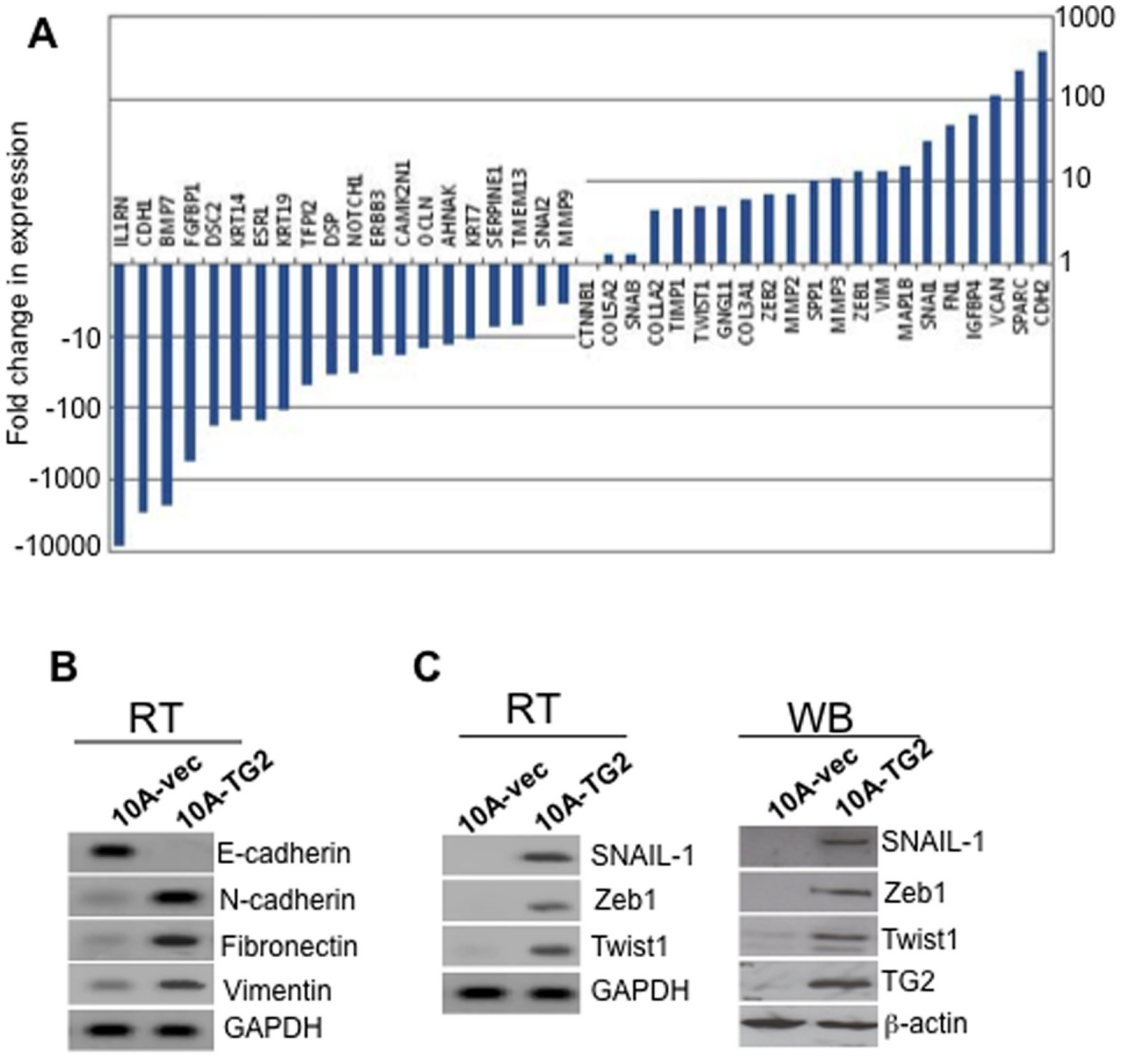
TG2 expression results in altered expression of Snail1, Twist1, Zeb1, and Zeb2. (**A**) Real time RT-PCR array showing relative changes in the expression of EMT-related genes in 10A-TG2 cells in comparison to 10A-Vec cells. Y-axis denotes the fold- expression and x-axis denotes the genes. The expression of GAPDH, β-actin and 18S ribosomal RNA was used to normalize variable template loading. (**B**) RT-PCR analysis for EMT-related transcripts was used to validate their expression in 10A-Vec and 10A-TG2 cells. (**C**) RT-PCR (left panel) and immunoblot (right panel) analysis was performed to validate the expression of transcription factors Snail1, Zeb1 and Twist1 in 10A-Vec and 10A-TG2 cells. Results shown are from a representative experiment repeated at least twice with similar results.

Induction of EMT is orchestrated by various transcription factors including Snail1, Slug, Twist, Zeb1, Zeb2, E12, E47 [20]. To determine which of these transcription factors are involved in TG2-induced EMT, we next performed transcriptional profiling of EMT-associated genes using the SABiosceinces' real-time PCR-based EMT array. Figure 2A shows the relative expression of EMT-associated genes in 10A-TG2 cells with respect to 10A-vec cells. The epithelial markers E-cadherin (*CDH1*), desmocollin 2, Krt 19, Krt14, Krt7, desmoplakin and occludin were downregulated whereas, the mesenchymal markers N-cadherin, versican, fibronectin, Sparc, and vimentin were upregulated. These results suggest that loss of E-cadherin and upregulation of N-cadherin (*CDH2*), fibronectin and vimentin in 10A-TG2 cells was regulated at the transcriptional level. To further validate these results, we performed RT-PCR for E-cadherin, N-cadherin, fibronectin and vimentin in 10A-vec and 10A-TG2 cells. Results shown in Figure 2B confirmed that TG2 expression was associated with a complete loss of E-cadherin transcript and increase in N-cadherin, fibronectin, and vimentin transcripts. Accordingly, 10A-TG2 cells showed a significant increase in the transcript levels of *Snail1, Zeb1, Zeb2*, and *Twist1* (Fig. 2A), the known transcriptional repressors of E-cadherin and inducers of N-cadherin, fibronectin, and vimentin. Similar results were obtained for Snail1, Zeb1, and Twist1 expression by RT-PCR (Fig. 2C, left panel) and western blot (Fig. 2C, right panel). These results suggested that TG2 expression induced transcriptional repression of E-cadherin and transactivation of fibronectin, N-cadherin, and vimentin by altering Snail1, Zeb1, Zeb2, and Twist1 levels.

### TG2-induced EMT confers invasiveness, drug resistance and tumorigenic phenotype

**Figure 3:**
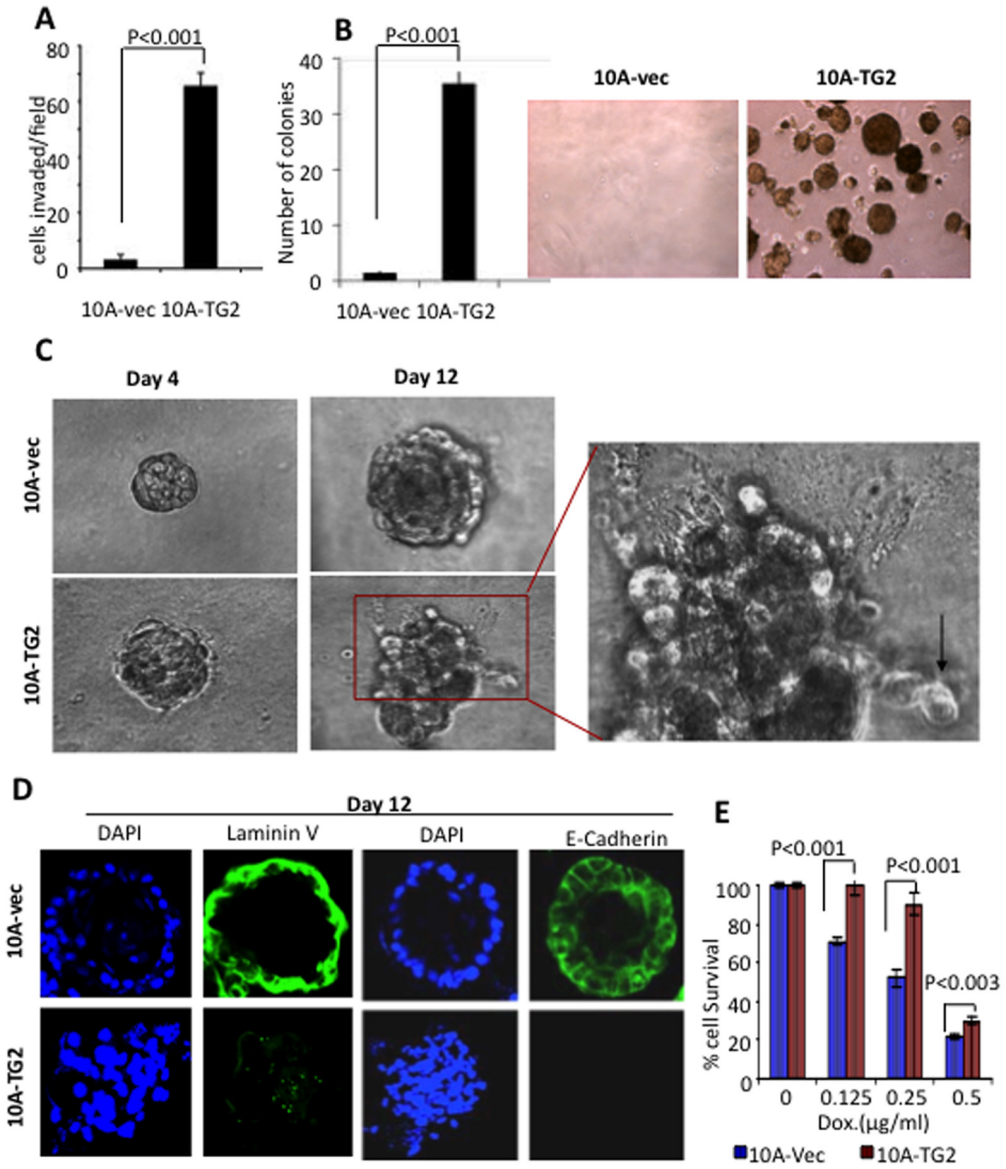
TG2-induced EMT confers invasiveness, drug resistance, and tumorigenic phenotype. (**A**) Transwell-Matrigel invasion assay was performed with 10A-Vec and 10A-TG2 cells. Cells that invaded though the Matrigel after 72 hr incubation were counted in five random microscopic fields under 20X magnification. Experiments were done three times in triplicate. Results shown are the average number of invading cells per field ± SEM. (**B**) Graph represents average number of colonies formed from three independent experiments ± SEM after 3 weeks' incubation of cells in soft agar. Images of colonies formed from a representative experiment after 3 weeks culture in soft agar are shown in right panel. Magnification 10X. (**C**) Phase-contrast images of acinar structures (4 and 12 days) formed as a result of 10A-Vec and 10A-TG2 cell culture in Matrigel-coated glass-slide chambers for indicated time periods. Inset, amplified view of individual MCF10A-TG2 cells invading the surrounding Matrigel (indicated by the arrow). (**D**) Loss of basement membrane integrity and cell-to-cell adhesion in 10A-TG2 acini. MCF10A cells were cultured in Matrigel-coated chambers for 12 days and immunostained for laminin V and E-cadherin (green) and DAPI (blue). Representative images from two independent experiments with similar results are shown. Magnifications 20X. (**E**) Sensitivity of 10A-Vec and 10A-TG2 cells to doxorubicin. Quadruplicate wells in 96-well plates, containing 2,000 cells per well in 0.2 ml of the complete medium (10% FCS) were either left untreated or treated with indicated concentrations of doxorubicin. Two days after the treatment, viable cells remaining in wells were determined by MTS reduction test, and percent cell viability was calculated. Experiments were repeated at least three times with similar results. Bars, mean of quadruplicate values from a representative experiment; lines, SD.

Based on the biological contexts, EMT has been classified into three subtypes: Type 1 is associated with embryonic development and neither causes fibrosis nor induces invasive phenotype. Type 2 EMT is associated with wound healing, tissue regeneration, and organ fibrosis and does not induce the invasiveness. Type 3 or oncogenic-EMT is associated with cancer and is characterized by acquisition of invasive and metastatic functions [21]. Accordingly, TG2 expression was associated with increased invasiveness of 10A-TG2 cells compared to the 10A-vec cells (Figs. 3A). These results suggest that TG2-induced EMT is the Type 3 and is associated with the acquisition of invasive and metastatic potential. As a result, we next determined weather TG2-induced EMT would promote the growth of 10A-TG2 cells in soft agar – an *in vitro* surrogate measure of tumorigenicity [22]. Results shown in Fig. 3B demonstrated that 10A-TG2 cells could grow and form colonies in soft agar whereas, 10A-vec cells failed to survive under these conditions.

MCF10A is a non-transformed human mammary epithelial cell line and is an excellent *in vitro* model to study mammary gland development and breast cancer progression in 3D cultures. These cells form well-organized acinar structures that mimic the normal mammary end bud *in vivo* [23]. We used this 3D model to determine weather TG2-induced EMT can disrupt the organization of MCF10A cells. As shown in Fig. 3C, the 10A-vec cells grew into well-organized acinar-like structures with hollow lumens (Fig. 3C and D, top panel). Immunostaining of spheroids with anti-laminin V antibody revealed the presence of continuous and well-defined basement membrane surrounding the acini with apicobasal polarization of cells (Fig. 3D). Similarly, immunostaining of spheroids for E-cadherin further confirmed the apico-basal polarization with cell-to-cell contact in acinar structures (Fig. 3D).

The 10A-TG2 cells, in contrast, demonstrated severe disruption of acinar architecture, characterized by irregular spheroid growth and no lumen formation (Figs. 3C and 3D, lower panels). A distinct feature of 10A-TG2 sheroids was the gain of invasive function; many cells escaped from the acini and invaded the surrounding matrix (Fig. 3C, inset). Similarly, immunostaining for laminin V and E-cadherin revealed diffused basement membrane formation and complete loss of E-cadherin in10A-TG2 acini (Fig. 3D). Similar changes in growth pattern and organization of acinar structure were observed in TG2-expressing MCF-12A cells, when grown in 3D culture (Supplemental Fig. S2). These results further supported the ability of TG2 to promote invasive potential in MECs. Several lines of evidence suggest that tumor cells undergoing EMT, become resistant to chemotherapy and conversely tumor cells selected for drug resistance exhibit EMT phenotype [17]. Therefore, we next determined weather TG2-induced EMT in MCF-10A cells could confer resistance to chemotherapeutic drug. Results shown in Fig. 3E demonstrated that 10A-TG2 cells were relatively more resistant to doxorubicin-induced cell death compared to the10A-vec cells. Overall, these results suggest that TG2-induced EMT promotes invasiveness, drug resistance and anchorage-independent growth of MECs.

### TG2 is a downstream mediator of TGF-β-induced EMT

**Figure 4:**
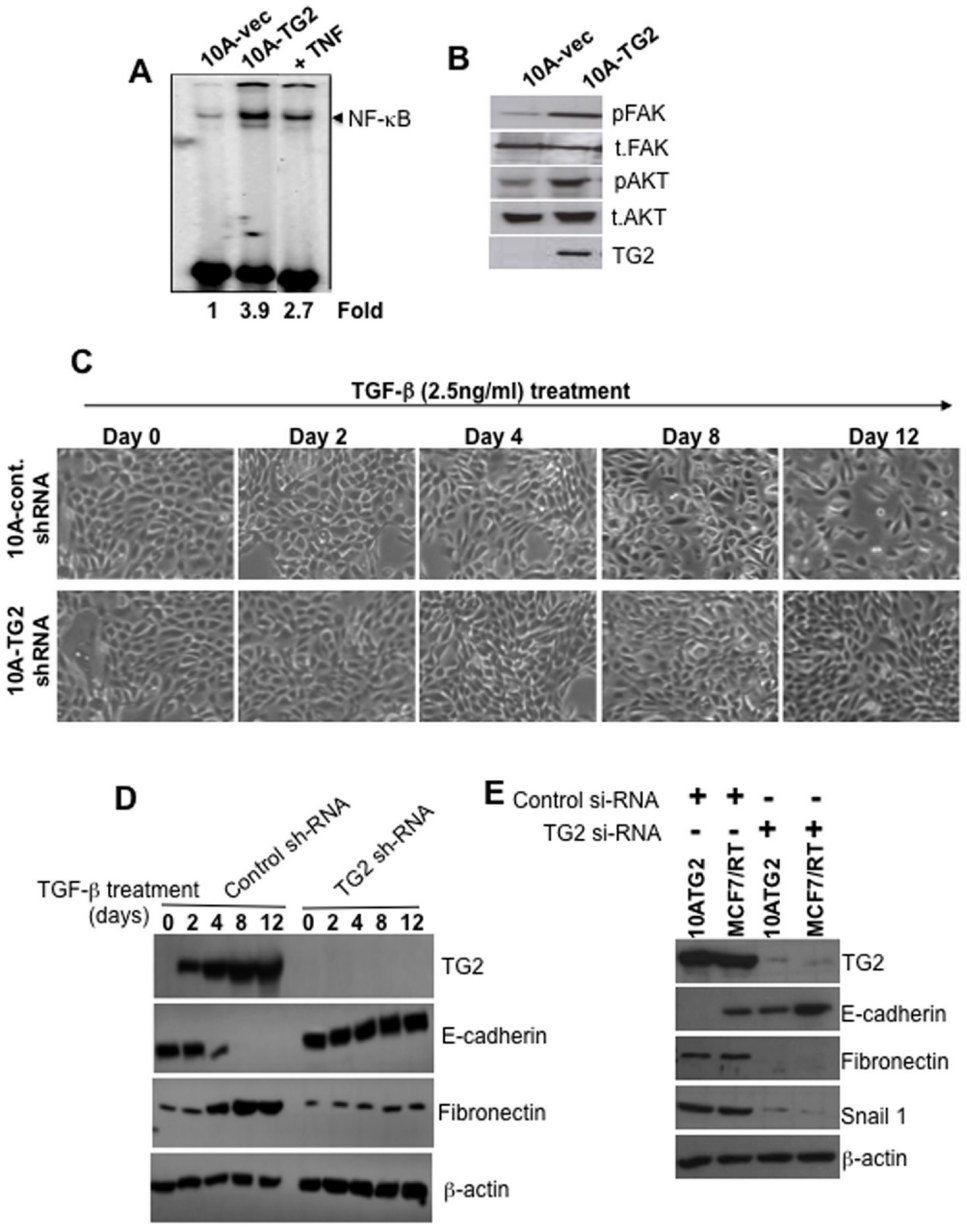
TG2 is a downstream mediator of TGF-β-induced EMT. (**A**) Supershift assay for NF-κB activity using EMSA with the nuclear extracts prepared from indicated cells. Nuclear extracts were incubated with an anti-p65 antibody and anti-p50 antibody, or nonradioactive (cold) or mutant NF-κB oligonucleotides and examined for DNA binding. Nuclear extract of KMB cells treated with TNF-α (+TNF) were used in parallel as positive control. (**B**) Immunoblot analysis showing the level for pAKT(S473) and pFAK(Y397) in 10A-Vec and 10A-TG2 cells. (**C**) Phase-contrast images of MCF10A cells transfected with control-shRNA or TG2-shRNA and incubated with 2.5 ng/ml recombinant TGF-β for indicated time periods. After 8 and 12 days of TGF-β-treatment MCF10A-control-shRNA cells showed mesenchymal morphology but not the TG2-shRNA transfected cells. (**D**) Immunoblot analysis for expression of TG2, E-cadherin and fibronectin in MCF10A-control-shRNA and MCF10A-TG2-shRNA cells in response to TGF-β treatment at indicated time points. (**E**) Downregulation of endogenous (MCF-7/RT) or induced (MCF10A-TG2) TG2 by siRNA resulted in loss of fibronectin (mesenchemyal marker) and upregulation of E-cadherin (epithelial marker) expression. Results shown are from a representative experiment repeated twice with similar results.

Because the EMT gene signature of 10A-TG2 cells (Fig. 2A) closely matched the TGF-β-induced EMT gene signature [24], we next determined whether TG2-induced EMT involved TGF-β signaling. For this purpose, we checked the expression of TGF-β receptor-1 and -2 and phospho-smad 2 and 3 in 10A-vec and 10A-TG2 cells. Interestingly, we did not observe any change in receptors level or phosphorylation status of smad-2 or samd-3 (Supplemental Fig. S3), suggesting that TG2-induced EMT is independent of TGF-β signaling. The progression of the EMT program is regulated by a series of intracellular signaling molecules, such as NF-κB, MAPK, PI3K, Akt, RhoB, Ras, and c-Fos as well as by cell-surface proteins, such as β4 integrin, α5β1 integrin, and αVβ6 integrin [25]. Previous studies have shown that TG2 expression results in constitutive activation of NF-κB [13], [26]. Therefore, next we determined the status of NF-κB activity in 10A-vec and 10A-TG2 cells. Results shown in Fig. 4A reveled almost 4-fold increase in NF-κB activity in 10A-TG2 cells compared with the 10A-vec cells. These results suggest that TG2-induced activation of NF-κB may be responsible for transcriptional regulation of *Snail1* and induction of EMT in 10A-TG2 cells. Indeed, TG2 was recently shown to associate with NF-κB for its recruitment to the promoter sequence of *Snail* and leading to its transcriptional regulation [27].

We also determined the status of pFAK and pAkt, the other known mediators of EMT [28], in MCF10A sublines. Results shown in Fig. 4B demonstrated the constitutive activation of both FAK and Akt in 10A-TG2 cells. These results suggest that TG2-mediated activation of FAK, Akt and NF-κB may play a role in driving MCF10A cells into EMT by regulating *Snail, Zeb1*, and *Twist* genes expression. Because TGF-β can induce TG2 expression [29], we next determined whether TG2 expression is required for TGF-β to induce EMT. MCF10A cells stably transfected with TG2-shRNA and treated with TGF-β showed no or minimal change in their morphology (Fig. 4C). In contrast, MCF10A-control shRNA-transfected cells under similar conditions, revealed progressive change in their morphology starting as early as 4 days post-TGFβ treatment. After 12-days of treatment, they appeared elongated and showed mesenchymal appearance (Fig. 4C). Western blot analysis for epithelial markers in TGF-β-treated MCF10A-control-shRNA cells revealed downregulation of E-cadherin on day 4 with complete loss by day 8 of treatment. No such change in E-cadherin level was evident in TG2shRNA-transfected cells even after 12 days of treatment. Similarly, fibronectin expression in response to TGFβ treatment was found upregulated but only in MCF10A-control shRNA cells (Fig. 4D). Similarly, downregulation of endogenous (in MCF-7/RT cells) or induced TG2 (in MCF-10A/TG2 cells) reversed the EMT process (mesenchymal-to-epithelial) as revealed by increase in E-cadherin and decrease in fibronectin expression (Fig. 4E). These results clearly implied that TG2 is a downstream mediator of TGF-β-induced EMT.

### TG2-induced EMT promotes stem cell-like phenotype

**Figure 5:**
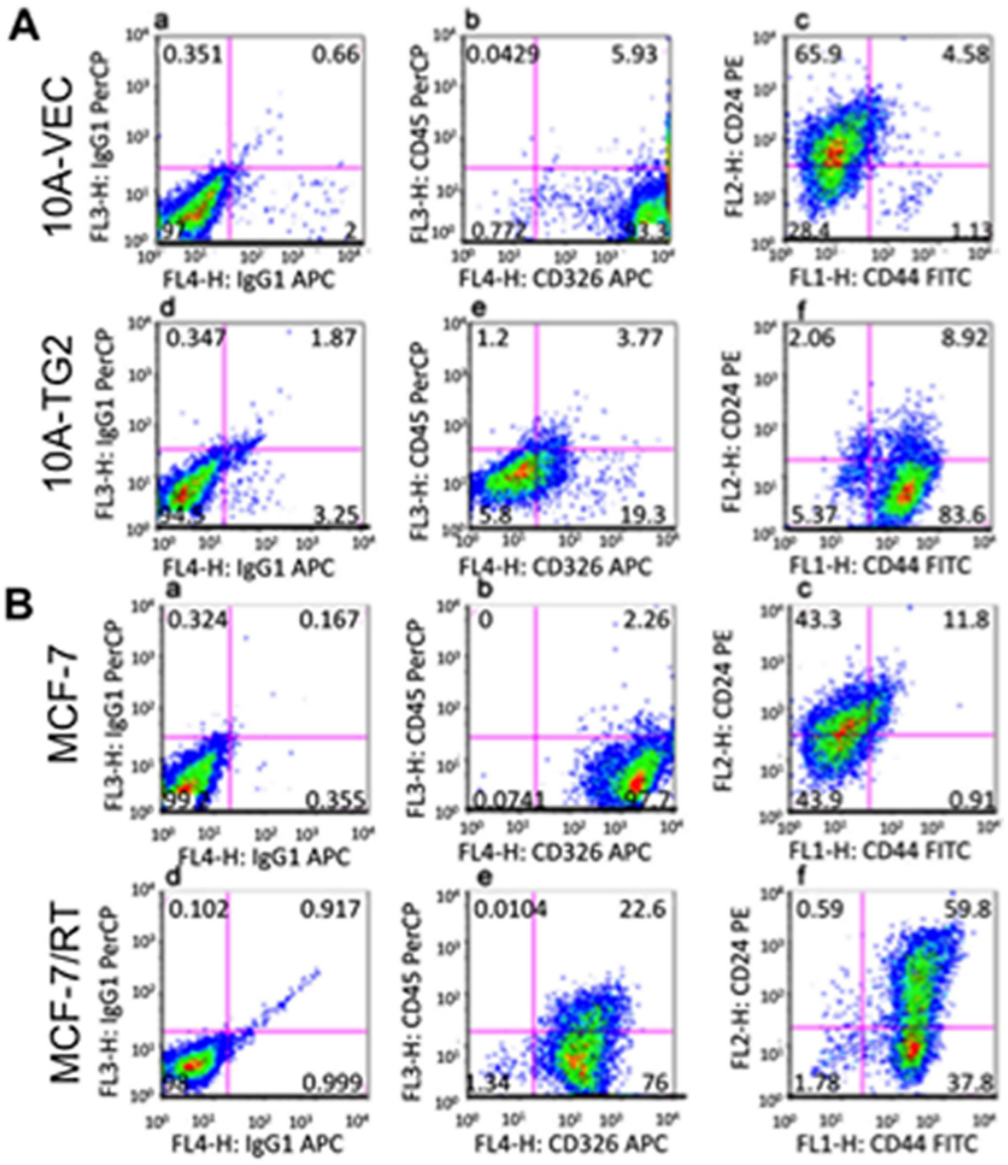
TG2-induced EMT promotes stem cell-like phenotype. FACS analysis of cell surface markers CD326, CD45, CD44 and CD24 in MCF10A (**A**) and MCF-7 (**B**) breast epithelial cells, expressing exogenous (10A-TG2) or endogenous (MCF-7/RT) TG2. 10A-Vec and MCF-7 cells, which lack TG2 expression, served as controls. Results shown are from a representative experiment repeated multiple times on different occasions.

Based on recent reports that induction of EMT results in acquisition of stem cell-like characteristics and that EMT and stem cells share some common molecular links in breast epithelial cells [30], next we determined whether TG2-induced EMT could induce a stem cell state in MCF10A cells. We used flow cytometric analysis to determine the expression of CD44^high^/CD24^-/low^ phenotype in 10A-TG2 and 10A-vec cells. The CD44^high^/CD24^-/low^ has been used as a marker to isolate stem cells from normal and cancerous mammary epithelial cells [31]. Results from a representative experiment shown in Fig. 5A revealed that significantly more 10A-TG2 cells expressed CD44^high^/CD24^-/low^ stem cell markers. Consistent with these results was the observation that CD326 antigen, an epithelial marker, was significantly downregulated in 10A-TG2 cells. To further validate these results, we next analyzed the CD44^high^/CD24^-/low^ expression in doxorubicin-resistant MCF-7/RT breast cancer cells [19]. The constitutive expression of TG2 in MCF-7/RT cells was associated with a similar enrichment of CD44^high^/CD24^-/low^ cell population compared to the parental drug-sensitive and TG2-deficient MCF-7 cells (Fig. 5B). However, as the MCF-7/RT subline represents a small subclone of MCF-7 cells, association between TG2 expression and CD44^high^/CD24^-/low^ phenotype needs to be further validated. Nevertheless, TG2 expression in yet another mammary epithelial cell line (MCF12A), showed a similar enrichment in CD44^high^/CD24^-/low^ expressing cells compared to the control vector-infected cells (Supplemental Fig. S4). Taken together, these results suggest a novel TG2-regulated pathway that could confer EMT and stem cell-like phenotype in mammary epithelial cells.

## Discussion

In this study, we show that aberrant expression of TG2 plays a critical role in promoting the EMT and EMT-dependent processes in epithelial cells. Thus, stable expression of TG2 in mammary epithelial cells was associated with increased invasion, loss of cell polarity, increased cell survival, anchorage-independent growth and resistance to chemotherapy. Our study provides first evidence on the functional significance of previously observed increased TG2 expression in drug resistant and metastatic tumors [2], [3], [11], [19], [26], [32].

Many recent reports have demonstrated elevated expression of TG2 in multiple metastatic tumors and those resistant to chemotherapy [2], [3], [11], [33]–[36] and its expression has been implicated in disease progression. [33], [35]. Thus, the observation that silencing of *TG2* gene expression due to hypermethylation of the CpG island that overlaps transcriptional and translational start site of *TG2* may explain relative sensitivity of primary tumors to chemotherapeutic drugs [36]. These findings suggest an interconnecting link between TG2 expression and progression of metastatic and drug-resistant cancer.

TG2 is structurally and functionally a complex protein. Depending on the cellular context, it can promote or suppress tumor growth [33]. Anti-tumorigenic function of TG2 are mainly linked to its ability to irreversibly crosslink proteins, which requires the presence of high calcium (≥1 mM) and low GTP (≤9 µM). Therefore, under the physiological conditions intracellular TG2 predominantly exists as a catalytically inactive protein due to low calcium and inhibitory effect of GTP [37]. Recent understanding of the molecular pathways that govern the progression of primary tumors to metastatic disease, points to the EMT as a central stage. The EMT is considered to be an essential process for development of different tissues during embryogenesis [17]. Its reactivation in adults can be considered as a physiological event to control inflammatory response and heal damaged tissues. However, in pathological context, such as in tumors or during organ fibrosis, this healing response may act in a harmful manner and result in metastasis and organ failure. The evidence is accumulating that prolonged induction of EMT plays an important role in cancer progression by converting immobile epithelial cells into fibroblast-like cells with reduced intercellular adhesion, increased motility and invasive behavior of cells [15]–[18], [20], [21]. Our current findings that stable expression of TG2 promotes drug resistance and invasiveness in normal and transformed mammary epithelial cells by inducing EMT suggest that TG2 serves as a converging point in conferring drug resistance and metastatic phenotype in cancer cells. A similar correlation between TG2-induced EMT and metastasis was recently observed in ovarian cancer cells. [38]

Metastasis of primary tumors to distant sites involves many steps including, successful invasion, intravasation, survival in circulation, extravasation and colonization by the cancer cells. Many cancer cell types rely on the EMT program for successful execution of these steps [17]. In order for cancer cells to break away from neighboring cells and invade adjacent cell layers, these tumor cells must lose cell-cell adhesion and acquire motility. EMT causes loss of epithelial markers like E-cadherin, desmocollin, desmoplakin, and occluding (involved in the formation tight connection between them) which loosens the cell-cell adhesion and helps in the dissemination of cells. It can modulate other adhesion systems and trigger the remodeling of the actin cytoskeleton, leading to the mesenchymal phenotype and increased scattering and motility of carcinoma cells [39]. Acquisition of the mesenchymal phenotype has also been associated with invasive behavior [40]. Indeed we observed that TG2 induced EMT in mammary epithelial cells increases the invasive potential and disrupts the organized growth in 3D culture. Induction of EMT in both normal and pathological conditions is choreographed by a set of EMT inducing transcription repressors, such as Snail, Slug, Twist, Zeb1, and Zeb2. Apart from inducing the EMT program these transcription factors also confer resistance to cell death, including resistance to chemotherapeutic drugs [17]. We found upregulation of some of these transcription factors (Snail1, Twist1, Zeb1, Zeb2) in TG2 expressing cells that was associated with increased resistance to doxorubicin. These observations suggest that TG2-induced EMT promotes drug resistance, an important trait of metastatic cancer cells.

Once detached form the original niche transformed epithelial cells need to acquire autonomy from growth regulatory mechanisms, which could give them the survival advantage. Normal epithelial cells die due to anoikis when detached form the neighboring cells. EMT promotes anchorage-independent growth in transformed mammary epithelial cells [30], a property that generally distinguishes transformed (tumorigenic) from normal (nontumorigenic) cells [22]. In this study we found that TG2-induced EMT promotes the anchorage-independent growth and protects mammary epithelial cells from death. TG2 is known to rescue cells from anoikis [41], which may explain the ability of MCF-10A-TG2 cells to grow in anchorage-independent manner. After successful invasion, intravasation, survival in circulation, and extrvasation, transformed epithelial cells must survive and colonize in the hostile environments of the foreign tissue. This process involves the growth of micrometastases into macroscopic metastases. Recently, it has been proposed that EMT can enable cancer cells not only to disseminate but also to acquire the ability of self-renewal by inducing a stem cell state [30]. In line with these observations, our initial results suggested that TG2-induced EMT in mammary epithelial cells could confer a stem cell-like phenotype (CD44^high^/CD24^-/low^). Currently we are determining the stem cell characteristics of TG2-transfected MCF10A and MCF12A cells by establishing their ability to self-renewal and to differentiate into multiple lineages.

**Figure 6:**
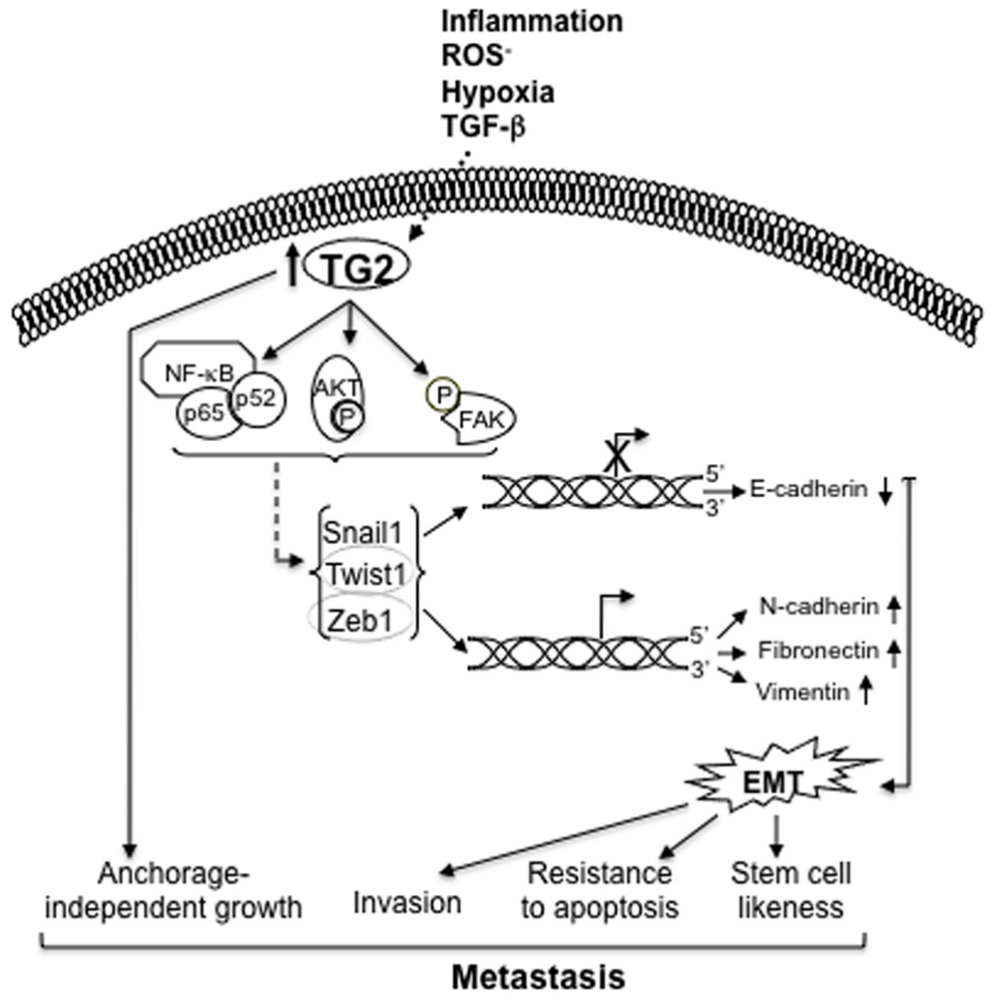
Schematic of TG2-indued pathways involved in promoting the metastatic phenotype. Inflammatory signals such as generation of ROS^-^, hypoxia, or TGF-β induce the expression of TG2 in epithelial cells. Induction of TG2 results in constitutive activation of AKT, FAK and NF-κB, which can lead to the transcriptional regulation of Snail1, Zeb1, and Twist. Expression of these transcription factors represses the E-cadherin and induces the expression of fibronectin, N-cadherin and vimentin. These changes result in transformation of immotile epithelial cells to motile mesenchymal cells resulting in altered cell-cell (homotypic) and cell-ECM (heterotypic) interactions and offers metastatic niche to the cells in terms of increased invasiveness, survival and self-renewing capacity by conferring stem cell-like phenotype. TG2 expression also promotes anchorage-independent growth.

While studies presented here clearly demonstrate the importance of TG2 in promoting the EMT in mammry epithelial cells, we are still working on the molecular intricacies through which TG2 modulates these functions. Previous reports as well as the current study demonstrate that aberrant expression of TG2 in epithelial cells results in constitutive activation of FAK, Akt, and NF-κB [10], [13], [26]. These pathways are known to be intimately involved in regulation of EMT, conferring drug resistance, and promoting metastasis [18], [21], [42]. For example, activated NF-κB is considered to be a hallmark of many advanced-stage tumors [43], [44]. Thus, constitutively active NF-κB is implicated to play a role in resistance to death-inducing stimuli, including chemotherapeutic agents [45] and to promote metastasis by inducing EMT [42]. The NF-κB-induced EMT has been attributed to the increased stability of Snail due to increased synthesis of ICOP9 signolosome 2, which blocks the ubiqutination and subsequent degradation of Snail [46]. In another report, constitutive activation of NF-κB in MCF10A cells was found to induce the EMT as a result of increased expression of Zeb1 and Zeb2 [47]. Based on these observations it is reasonable to believe that TG2-induced EMT may result from constitutive activation of NF-κB and subsequent increase in Snail, Zeb1, and Zeb2 expression, as observed in this study. Indeed, TG2 was recently shown to associate with NF-κB for its recruitment to the promoter sequence of Snail and leading to its transcriptional regulation [27]. Similarly TGF-β, which is considered to be a potent inducer of EMT both in normal and pathological conditions [17], [24], can cross-talk with TG2. Thus, TGF-β can induce TG2 expression [48], [49] and TG2 can activate TGF-β [50]. Although, we did not observe any activation of TGF-β signaling in TG2-transfected MCF10A cells, nevertheless, TGF-β failed to induce EMT in absence of TG2, suggesting that TG2 is an important downstream mediator of TGF-β-induced EMT. Although additional studies are needed to further validate tumorigenic potential of MCF10A-TG2 cells, our *in vitro* data clearly support that stable expression of TG2 is sufficient to induce metastatic phenotype. Based on these results, we hypothesize a model that aberrant expression of TG2 contributes to the transformation of primary breast cancer to metastatic capabilities (Fig. 6). These results also support our belief that TG2 is a promising therapeutic target for drug-resistant and metastatic breast cancer.

## Methods

### Cell lines and vectors

The immortalized human mammary epithelial cells (MCF10A and MCF12A) and breast cancer cell line MCF7 and MCF7-RT were maintained as previously described [23], [19]. TG2 gene was subcloned into pCDH lentiviral vector (System Biosciences, Frederick, MD) from pcDNA3.1-TG2 vector (kindly provided by Dr. Gail Johnson, University of Rochester, NY). Control ShRNA and TG2 shRNA lentiviral particles were purchased from Santa Cruz Biotechnology (Santa Cruz, CA). 10A-Vec and 10A-TG2 cells were made by retroviral infection. Retroviral infection of cells and their 3D culture were done as previously described [23]. Stable clones were selected against 800 ng/ml puromycin. Multiple stable clones were used to rule out potential clonal effects. All experiments were performed between passage 5 and 25. Lentivirus production and infection, and reagents used, are described in the Supplemental Procedures S1 and Table S2. For transient transfection TG2-specific siRNA and control siRNA were purchased from Qiagen (Valencia, CA) and MCF7/RT and 10A-TG2 cells were transfected as described earlier (3). For TGF-β treatment, MCF10A-control-shRNA and MCF10A-TG2 shRNA cells were cultured in MCF10A medium and treated with 2.5 ng/ml of recombinant-TGFβ1 (kindly provided by Dr. Bharat Aggarwal, M. D. Anderson Cancer Center) for up to 12 days.

### Antibodies, Western Blotting, and Immunofluorescence

For Western blots, cells were lysed on ice in 50 mM Tris-HCl buffer, pH 7.5 containing 150 mM NaCl and 0.5% NP-40. Fifty micrograms of total protein from each sample were resolved on a 4%–12% SDS Bis-Tris-polyacrylamide gel with running buffer and transferred onto nitrocellulose membranes. The blots were then probed with various antibodies (details are given in Supplemental Table S1). Immunofluorescence staining of monolayer cell cultures and 3D culture was done as previously described [23].

### RNA Extraction, RT-PCR, and Quantitative RT-PCR

The detailed procedures for RNA extraction RT-PCR and primers sequences are described in the Supplemental Procedures S1. Quantitative RT-PCR for EMT-associated genes was done using SABiosciences (Frederick, MD) EMT-PCR Array according to the manufacturer's protocol.

### Invasion, Soft Agar Colony Formation, Cell viability assay and NF-κB activity

Invasion assay, cell viability assay to check the drug resistance and NF-κB activity assay were performed as described previously [3], [13], [19]. Soft agar assays were performed as described previously [22]. Cultures were photographed, and the colonies with diameters larger than 500 mm were counted using Image J software (previously NIH image).

### FACS Analysis

The anti-CD44 (clone G44-26), anti-CD24 (clone ML5), and anti-CD45 (clone 2D1) antibodies used for FACS analysis were obtained from BD Biosciences (San Jose, CA). The anti-CD326 (clone HEA-125) was obtained from Miltenyi Biotec (Auburn, CA). Briefly, for each cell line, 5×10^5^ cells were aliquoted into 2 tubes; tube 1 was stained with IgG isotype controls for FITC, PE, PerCP, and APC; tub e 2 was stained with anti-CD44-FITC, anti-CD24-PE, anti-CD45-PerCP, and anti-CD326-APC. Cells were incubated with appropriate antibodies at room temperature in dark for 30 minutes, and then washed with PBS. Cells were acquired by 4-color FACS Calibur (BD Biosciences), each sample was acquired 10,000 cells for analysis.

## Supporting Information

Procedures S1 (0.03 MB DOC)

Table S1 Antibodies used.
(0.05 MB DOC)

Table S2 Primers for RT-PCR.
(0.04 MB DOC)

Figure S1 Immunofluorescence showing the accumulation of stress fibers in MCF10A-TG2 cells.
(7.71 MB TIF)

Figure S2 Phase-contrast images of acinar structures (4 and 12 days) formed as a result of MCF12A-vec and MCF12A-TG2 cell culture in Matrigel-coated glass-slide chambers for indicated time periods.
(7.71 MB TIF)

Figure S3 Immunoblot showing the expression of TGF-β receptor I & II, pSmad-2, and -3 and total smad-2 and 3 in 10A-vec and 10A-TG2 cells.
(7.71 MB TIF)

Figure S4 Flow cytometric analysis of TG2-transfected (TG2) and vector-alone (-vec) transfected mammary epithelial cells (MCF12A) for CD44+/CD24-/low stem cell marker expression.
(7.71 MB TIF)
