# Supplementary material for: Tissue Transglutaminase Promotes Drug Resistance and Invasion by Inducing Mesenchymal Transition in Mammary Epithelial Cells
Source: PLoS One. 2010 Oct 12;5(10):e13390. doi: 10.1371/journal.pone.0013390 (PMC2953521; doi:10.1371/journal.pone.0013390)
Supplement: Procedures S1 — (0.03 MB DOC) [file pone.0013390.s001.doc]

**Supplementary procedures – S1**

**Lentivirus production and infection:** Lentiviral vector and its packaging vectors were transfected into 293T packaging cells by lipofectamine transfection. Briefly, 293T cells were aliquoted (1x10^6^) into 25 cm^2^ flask one day before transfection. Then the cells were transfected with 2.5 μg pCDH-TG2 vector, together with 1.875 μg of psPAX2 (packaging vector) and 0.625 μg of pMD2G (envelope vector). After 5 hr incubation, the transfection medium was replaced with fresh culture medium; 48 hr later, the lentivirus-containing medium was collected and centrifuged at 1500 rpm for 5 min to pellet the cell debris, the supernatant was passed through a 0.45-μm filter, and the target cells were infected with fresh lentivirus-containing medium (supplemented with 8 μg/ml Polybrene) for 48 hr. Similarly, TG2-shRNA and control shRNA lentivirous were infected to target cells. After 48 hr incubation, cells were subjected to puromycin selection.

**RNA extraction, RT-PCR and quantitative RT-PCR:** Total RNA was extracted using Quiagen mini-RNA isolation kit according to the manufacturer’s protocol. For RT-PCR, 2μg total RNA was reverse transcribed to cDNA using Superscript III First Strand Synthesis System (Invitrogen). An equivalent volume (2 μl) of cDNA was used as the template for PCR using gene-specific primers. Quantitative RT-PCR for EMT-associated genes was performed, using SAbiosciences EMT array. Relative change was calculated after normalization to GAPDH, β actin and 18s ribosomal RNA
