# Supplementary material for: Tissue Transglutaminase Promotes Drug Resistance and Invasion by Inducing Mesenchymal Transition in Mammary Epithelial Cells
Source: PLoS One. 2010 Oct 12;5(10):e13390. doi: 10.1371/journal.pone.0013390 (PMC2953521; doi:10.1371/journal.pone.0013390)
Supplement: Table S1 — Antibodies used. (0.05 MB DOC) [file pone.0013390.s002.doc]

| *Antigens* | *Catalog #* | *Source* | *Application* |
| --- | --- | --- | --- |
| TG2 | Cub7402 | Neomarkers | IB, IFS |
| E-cadherin | Sc-21791 | Santa Cruz Biotech | IB |
| E-cadherin | 610182 | BD Transduction Laboratories | IF |
| N-cadherin | Sc-59987 | Santa Cruz Biotech | IB |
| Fibronectin | sc-9068 | Santa Cruz Biotech | IB,IFS |
| vimentin | Sc-66001 | Santa Cruz Biotech | IB,IFS |
| β-catenin | 610154 | BD Transduction Laboratories | IB,IFS |
| β-actin | A2172 | Sigma-Aldrich | IB |
| Snail1 | Sc-28199 | Santa Cruz Biotech | IB |
| Twist1 | sc-15393 | Santa Cruz Biotech | IB |
| Zeb1 | Sc-81428 | Santa Cruz Biotech | IB |
| smad2 | 3122 | Cell signaling technology | IB |
| smad3 | 9513 | Cell signaling technology | IB |
| p-smad2 | 3104 | Cell signaling technology | IB |
| p-smad3 | 9520 | Cell signaling technology | IB |
| TGFβ Receptor I | 3712 | Cell signaling technology | IB |
| TGFβ Receptor II | 2518 | Cell signaling technology | IB |
| pAkt | 9271 | Cell signaling technology | IB |
| Akt | 4691 | Cell signaling technology | IB |
| pFAK | 611722 | BD Transductuion laboratories | IB |
| FAK | 610087 | BD Transductuion laboratories | IB |
| Laminin V | MAB1947 | Chemicon International | IFS |

IB: Immunoblot, IFS: Immunofluorescence staining
