# Supplementary material for: Tissue Transglutaminase Promotes Drug Resistance and Invasion by Inducing Mesenchymal Transition in Mammary Epithelial Cells
Source: PLoS One. 2010 Oct 12;5(10):e13390. doi: 10.1371/journal.pone.0013390 (PMC2953521; doi:10.1371/journal.pone.0013390)
Supplement: Table S2 — Primers for RT-PCR. (0.04 MB DOC) [file pone.0013390.s003.doc]

| GENES | Primers |
| --- | --- |
| E-cadherin | F: 5’-CAAAAGAACTCAGCCAAGTG-3’ |
|  | R :5’- TGGCGTCGGAACTGCAAAG-3’ |
| N-cadherin | F :5’-ACAGTGGCCACCTACAAAGG-3’ |
|  | R: 3’-CCGAGATGGGGTTGATAATG-5’ |
| fibronectin | F :5’-CAGTGGGAGACCTCGAGAAG-3’ |
|  | R :3’-TCCCTCGGAACATCAGAAAC-5’ |
| vimentin | F :5’-GAGAACTTTGCCGTTGAAGC -3’ |
|  | R :3’- GCTTCCTGTAGGTGGCAATC-5’ |
| Snail1 | F: 5’-TTCTTCTGCGCTACTGCTGCG-3’ |
|  | R:5’GGGCAGGTATGGAGAGGAAGA-3’ |
| Twist1 | F: 5’AGCTGAGCAAGATTCAGACCCTC-3’ |
|  | R: 5’-CCGTCTGGGAATCACTGTC-3’ |
| Zeb1 | F: 5’-CTGAAGAGGACCAGAGGCAG-3’ |
|  | R: 5’-CCCAGAACTGCGTCACATGTC-3’ |
| GAPDH | F: 5’TGGTATCGTGGAAGGACTCATGAC-3’ |
|  | R: 5’-ATGCCAGTGAGCTTCCCGTTCAGC-3’ |
