# Supplementary figures and images for: Tissue Transglutaminase Promotes Drug Resistance and Invasion by Inducing Mesenchymal Transition in Mammary Epithelial Cells
Source: PLoS One. 2010 Oct 12;5(10):e13390. doi: 10.1371/journal.pone.0013390 (PMC2953521; doi:10.1371/journal.pone.0013390)

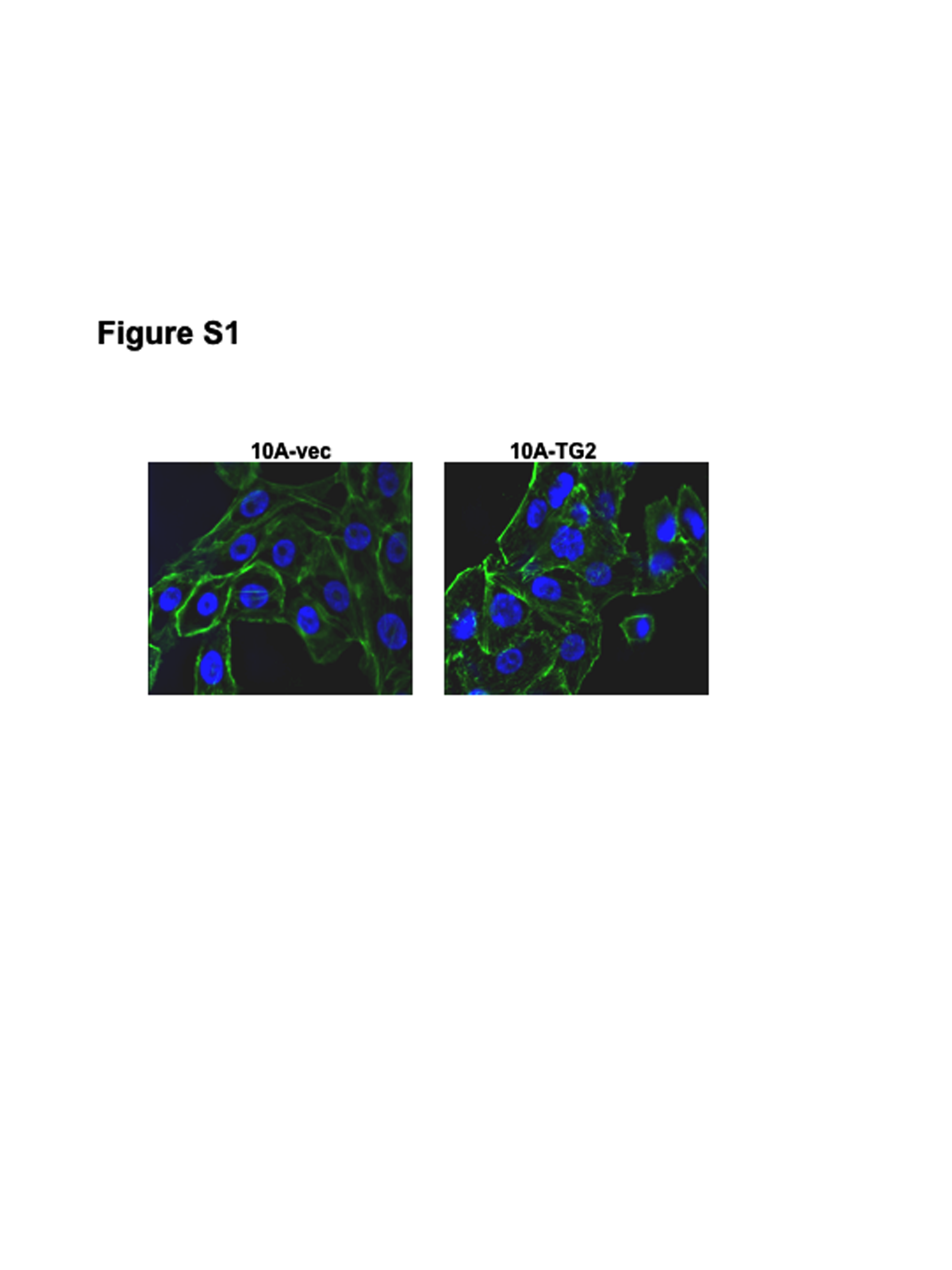

Supplement: Figure S1 — Immunofluorescence showing the accumulation of stress fibers in MCF10A-TG2 cells. (7.71 MB TIF) [file pone.0013390.s004.tif]

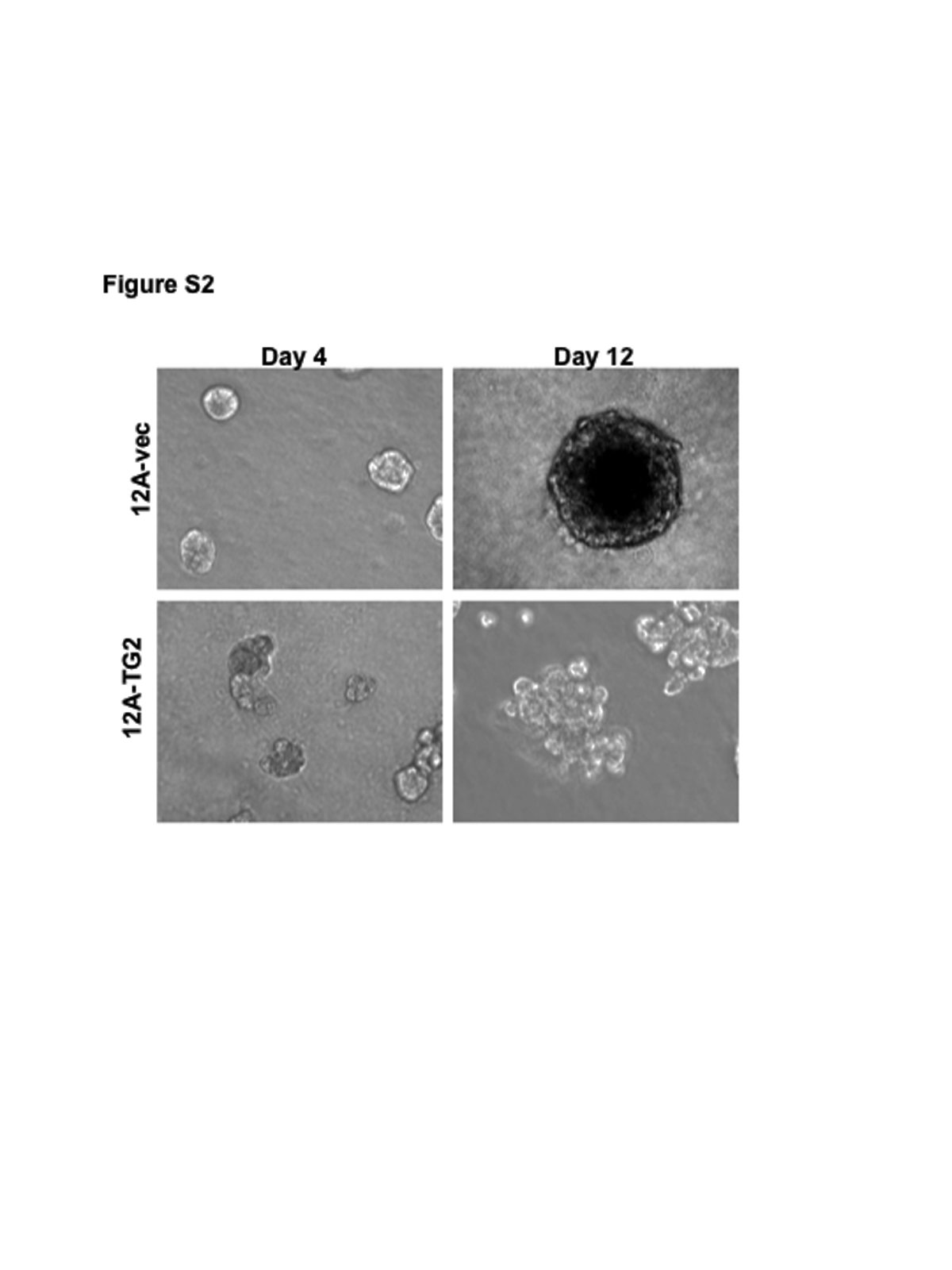

Supplement: Figure S2 — Phase-contrast images of acinar structures (4 and 12 days) formed as a result of MCF12A-vec and MCF12A-TG2 cell culture in Matrigel-coated glass-slide chambers for indicated time periods. (7.71 MB TIF) [file pone.0013390.s005.tif]

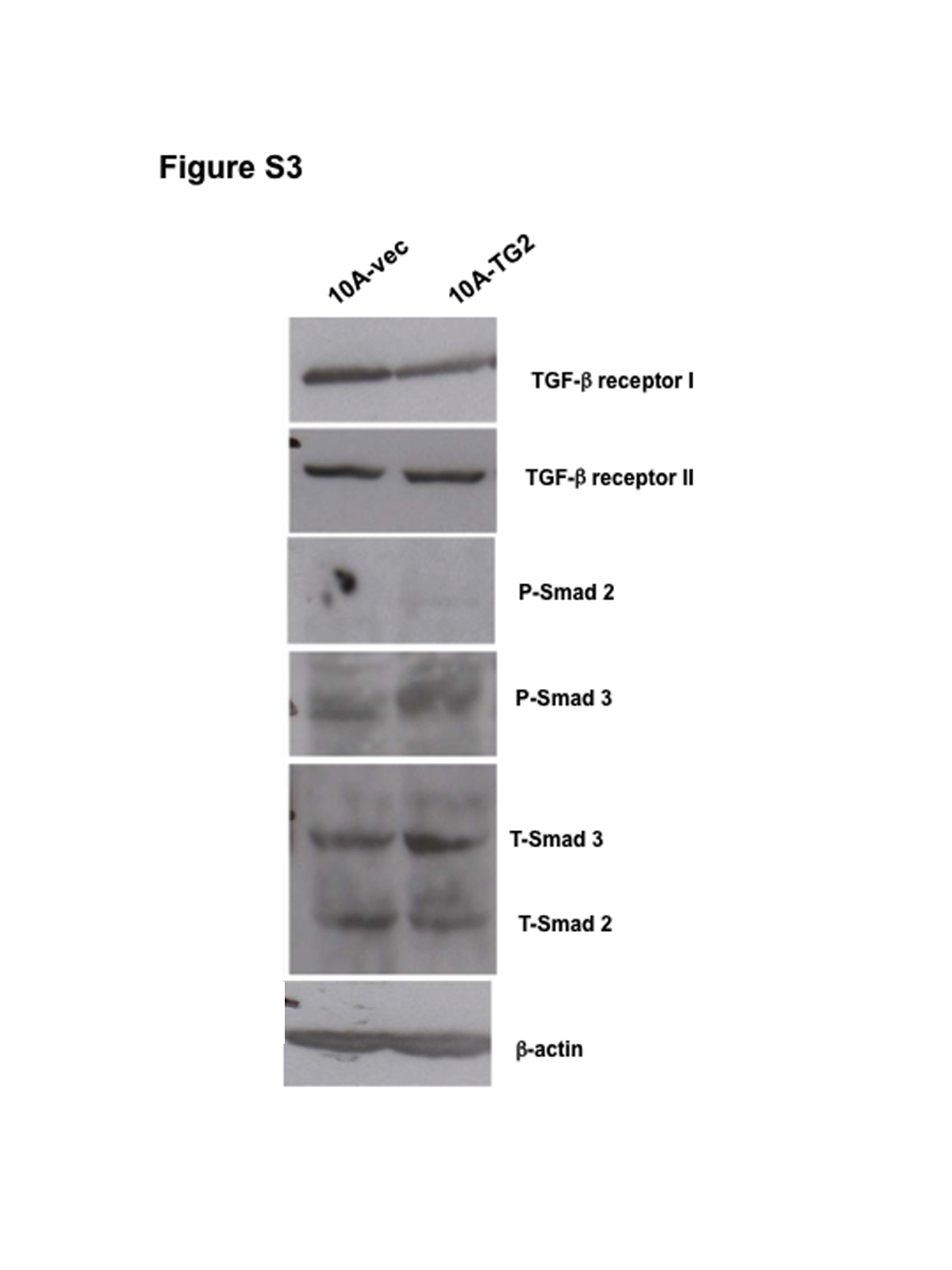

Supplement: Figure S3 — Immunoblot showing the expression of TGF-β receptor I & II, pSmad-2, and -3 and total smad-2 and 3 in 10A-vec and 10A-TG2 cells. (7.71 MB TIF) [file pone.0013390.s006.tif]

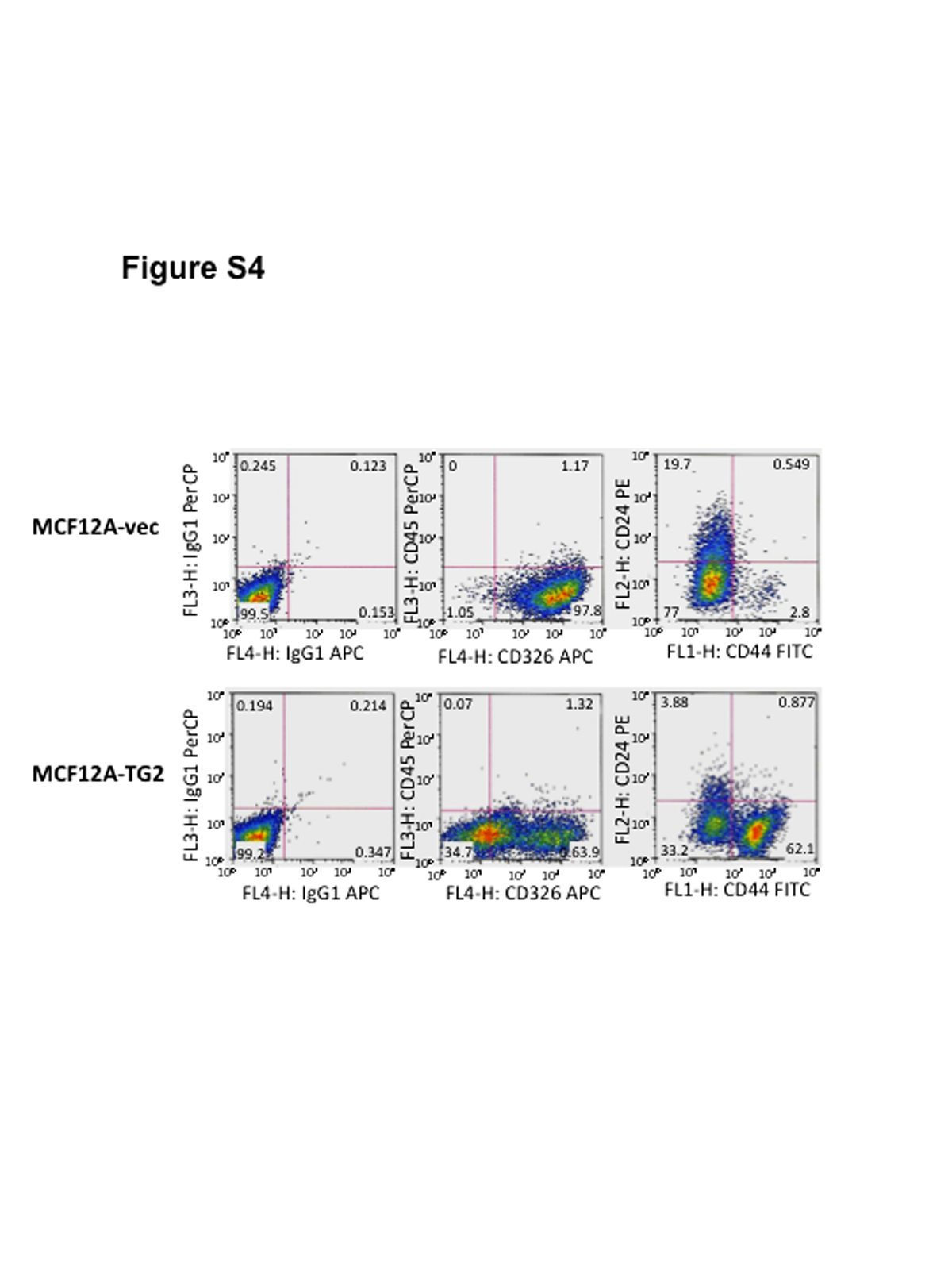

Supplement: Figure S4 — Flow cytometric analysis of TG2-transfected (TG2) and vector-alone (-vec) transfected mammary epithelial cells (MCF12A) for CD44+/CD24-/low stem cell marker expression. (7.71 MB TIF) [file pone.0013390.s007.tif]
